# Correction: Ecuador's Mangrove Forest Carbon Stocks: A Spatiotemporal Analysis of Living Carbon Holdings and Their Depletion since the Advent of Commercial Aquaculture

**DOI:** 10.1371/journal.pone.0124185

**Published:** 2015-04-24

**Authors:** The PLOS ONE Staff

There are superscript errors in the first, second, third, and fourth equations under the subheading Estimating Mangrove Carbon in the Methods section. Please view the complete, correct equations here:

$$CC{*}_{i}(t.ha^{-1})=(M_{it}\;(.464\;(373.273–8.486|Lat|))$$ Equation 1

CC* = combined carbon, *t* = tonnes, *ha* = hectare, M_*it*_ = Mangrove density at grid location *i* and time slice *t* on a scale of 0 to 1, |*Lat*| = absolute latitude.

$$CC_{i}(t.ha^{-1})=(M_{it}\;(.464\;(543.27–13.269|Lat|))$$ Equation 2

*CC* = combined carbon, *t* = tonnes, *ha* = hectare, M_*it*_ = Mangrove density at grid location *i* and time slice *t* on a scale of 0 to 1, |*Lat*| = absolute latitude.

$$CC{’}_{i}(t.ha^{-1})=(M_{it}\;(.464\;(\mathrm{0.90}*BM_{R}+\mathrm{0.05}*BM_{B}+\mathrm{0.05}*BM_{W})))$$ Equation 3

*CC’* = combined carbon, *t* = tonnes, *ha* = hectare, M_*it*_ = Mangrove density at grid location *i* and time slice *t* on a scale of 0 to 1, *BM*_*(s)*_ = combined biomass of species (*s*), _R_ = *Rhizophora mangle*, _B_ = *Avicennia germinans*_, W_ = *Laguncularia racemosa*

CC^i(t.ha−1)=(Mit(.464(nMRit*BMR+nMBit*BMB+nMWit*BMW))) Equation 4

*CC^* = combined carbon, *t* = tonnes, *ha* = hectare, M_*it*_ = Mangrove density at grid location *i* and time slice *t* on a scale of 0 to 1, *nM*_(*s*)it_ = normalized presence likelihood of species (*s*) at grid location *i* and time slice *t*, *BM*_*(s)*_ = combined biomass of species (*s*), _R_ = *Rhizophora mangle*, _B_ = *Avicennia germinans*_, W_ = *Laguncularia racemosa*.

Additionally, CMB is incorrectly written as CMD in the fourth sentence of the third paragraph under the subheading Estimating Mangrove Carbon in the Materials and Methods section. The correct sentence is: This conversion from CMB to CC* was conducted using the mangrove biomass to carbon ratio of *1*: *0*.*464* [7, 32].

In addition, the unit ha^-1^ is incorrectly written as ha-^1^ within the first two sentences of the second paragraph of the Introduction.
